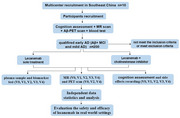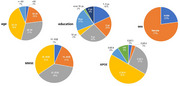# Clinical evaluation of the safety and efficacy of lecanemab treatment in early AD patients in Southeast China (a multicenter cohort study)

**DOI:** 10.1002/alz70857_101471

**Published:** 2025-12-25

**Authors:** Guoping Peng, Yan Sun

**Affiliations:** ^1^ The First Affiliated Hospital, Zhejiang University School of Medicine, Hangzhou, Zhejiang, China; ^2^ Zhejiang University School of Medicine, Hangzhou, Zhejiang, China

## Abstract

**Background:**

This multicenter study aims to describe efficacy and clinical safety of early Alzheimer's disease (AD) patients receiving lecanemab, and examine the dynamic changes of biomarker characteristics based on blood in real‐world settings.

**Methods:**

This study is a population‐based prospective cohort study currently enrolling 156 participants across ten centers in Southeast China, and the final number of enrolled early AD patient is 200. Demographics included age, sex, race/ethnicity, region and urbanicity, and baseline clinical characteristics were recorded. All enrolled AD patients were diagnosed according to the NIA‐AA 2024 criteria with AV45 PET scan. Cognitive scales assessment, blood samples collection, multiple sequences MR scan and AV45 PET scan were carried out before the first infusion of lecanemab, and would be finished on four observation time points. All adverse reactions include infusion reactions and amyloid related imaging abnormalities (ARIA) are also recorded. After collection, blood samples are analyzed at a central laboratory to measure plasma biomarkers levels. A flowchart illustrating the study process is shown in Figure 1.

**Results:**

Among the 156 patients enrolled at baseline, a total of 118 individuals received lecanemab treatment were continued infusion over three months, including 71 females and 47 males. As for the baseline, about 70% were aged 60‐79 years, 74% had 6 years or more of education, 8% were APOE4 homozygotes, 53% were APOE4 heterozygotes, with an average MMSE score of 21.9 ± 4.6 and an average CDR sum of box score of 3.9 ± 1.2 (see Figure 2). As for the 118 subjects that finished 3 month of follow‐up, 23 patients reported extremely mild to mild infusion reactions, ten patients experienced ARIA reactions, and three of them are symptomatic and two of them have stopped infusion as a result. The necessary data would be collected and analyzed in the follow up time points.

**Conclusions:**

This study will provide crucial data on the effectiveness and safety of commercially available lecanemab in amyloid‐positive early AD patients in Southeast Chinese population. The findings will further testify the potential ability of blood biomarkers in the prediction of therapeutic effects.